# A simple method to account for skin dose enhancement during treatment planning of VMAT treatments of patients in contact with immobilization equipment

**DOI:** 10.1002/acm2.12394

**Published:** 2018-06-22

**Authors:** James Rijken, Tanya Kairn, Scott Crowe, Luis Muñoz, Jamie Trapp

**Affiliations:** ^1^ Genesis Care Flinders Private Hospital Bedford Park SA Australia; ^2^ Queensland University of Technology Brisbane QLD Australia; ^3^ Royal Brisbane and Women's Hospital Brisbane QLD Australia

**Keywords:** skin, immobilization, SBRT, SABR, VMAT

## Abstract

**Purpose:**

The ability to accurately predict skin doses and thereby design radiotherapy treatments that balance the likelihood of skin reactions against other treatment objectives is especially important when hypofractionated prescription regimes are used. However, calculations of skin dose provided by many commercial radiotherapy treatment planning systems are known to be inaccurate, especially if the presence of immobilization equipment is not accurately taken into account. This study proposes a simple method by which the accuracy of skin dose calculations can be substantially improved, to allow informed evaluation of volumetric modulated arc therapy (VMAT) treatment plans.

**Method:**

A simple method was developed whereby dose calculation is split into grid regions, each with a correction factor which determines MU scaling for skin dose calculation. Correction factors were derived from film measurements made using a geometrically simple phantom in partial contact with a vacuum immobilization device. This method was applied to two different test treatments, planned for delivery to a humanoid phantom with a hypofractionated stereotactic body radiotherapy technique, and results were verified using film measurements of surface dose.

**Results:**

Compared to the measured values, calculations of skin dose volumes corresponding to different grade tissue reactions were greatly improved through use of the method employed in this study. In some cases, the accuracy of skin dose evaluation improved by 76% and brought values to within 3% of those measured.

**Conclusion:**

The method of skin dose calculation in this study is simple, can be made as accurate as the user requires and is applicable for various immobilization systems. This concept has been verified through use on SBRT lung treatment plans and will aid clinicians in predicting skin response in patients.

## 1 INTRODUCTION

As radiotherapy treatment complexity and precision increases, so does the need to ensure that patients are in reproducible and stable positions during treatment delivery. Immobilization equipment (vac bags, thermoplastic masks, belts etc.) is increasingly being used during radiotherapy treatment and commercial radiotherapy treatment planning systems (TPS) are known to produce inaccurate calculations of surface dose from megavoltage photon beams,^1^, ^2^, ^3^, ^4^, ^5^, ^6^, ^7^ even without the involvement of immobilization equipment.

Immobilization equipment can produce a skin dose enhancement effect from megavoltage photon treatments due to the buildup of scattered and photo‐electrons.^8^ For example, skin dose from a 6 MV photon beam can be increased by up to 22% and 43% due to 2.5 and 10 cm thick vacuum bags, respectively.^9^, ^10^ Carbon fiber couch tops contribute significantly to skin dose for VMAT plans with one study reporting skin doses as high as 81% of the prescription dose in regions of couch involvement.^11^ Thermoplastic immobilization for breasts has been demonstrated to produce an increase in skin dose upwards of 62%.^12^ Overall, the dose enhancement effects of couches and immobilization equipment are well documented^13^, ^14^ but TPS‐specific tools of management thereof are not, especially for VMAT.

Undesired dose enhancements are difficult to avoid and are especially important when VMAT treatments are used due to the substantial proportion of arc delivery through couch, vac bag, or other immobilization systems^14^ — effects that may have traditionally been avoided through careful selection of beam arrangement in static gantry treatments. This is exacerbated when treatments are delivered via hypofractionated regimes such as stereotactic ablative body radiotherapy (SABR or SBRT) and stereotactic radiosurgery (SRS) due to the increased fractionated dose delivery to “early responding” skin.^15^ SBRT treatments have produced grade 3–4 skin reactions in lung cases^16^, ^17^ and grade 1–2 reactions in spine cases.^18^

Treatment planning systems cannot, of course, provide accurate predictions of skin dose if the effects of the immobilization systems are not included. Previous studies have attempted to account for the effects of immobilization equipment through several methods not limited to (a) creating an artificial bolus structure in the TPS,^19^ (b) contouring fixation devices/couch in the TPS either with an appropriate assigned density or according to their tissue‐equivalent thickness^20^, ^21^, ^22^ or (c) by including the immobilization devices within the patient's body contour.^1^, ^23^ The studies by Lee et al. and Chan et al. improved skin calculations from a pencil beam algorithm for IMRT beams through the second method.^20^, ^22^ It remains to be seen whether this method is useful in the Pinnacle TPS or in a VMAT setup where portions of the treatment arc are delivered just though vac bag and not couch. A recent study by Wang et al. used comparisons with Monte Carlo dose calculations to establish that the accuracy of skin dose calculations provided by the Eclipse™ TPS (Varian Medical Systems, Palo Alto, CA, USA) could be substantially improved using the simple method of enlarging the patient's body contour (which defines the extent of the dose calculation region), so that it extends 2 cm outside the skin in all directions and specifically includes the treatment couch and immobilization devices.^1^ The validity of this solution for treatment planning systems other than Eclipse is yet to be established.

Producing radiotherapy (especially SBRT) treatments that provide adequate skin sparing while also achieving all other treatment goals (PTV coverage, OAR sparing) is difficult when treatment planning systems are unable to provide accurate predictions of skin dose. In this study, we evaluate the uncertainties in the skin dose calculations provided by the Pinnacle^3®^ 9.10 (Koninklijke Philips N.V., Amsterdam, the Netherlands) TPS, with and without immobilization equipment in contact with the skin and develop a simple method by which those uncertainties could be minimized by measuring and accounting for the effects of immobilization systems during dose calculations for VMAT treatment plans.

## 2 MATERIALS AND METHOD

### 2.A Measurement of skin dose correction factors

**Figure 1:**
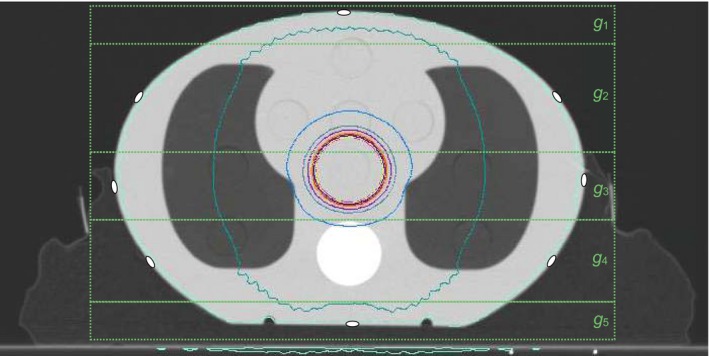
Vac bag immobilized CIRS phantom planned with a 4 × 4 cm^2^ 360° reference arc with *g*_*i*_ defined and film measurement locations indicated on the surface.

A CIRS 002LFC thorax phantom (Computerized Imaging Reference Systems, Inc., Norfolk, VA, USA) was CT scanned on a SOMATOM Emotion 6 CT scanner (Siemens AG, Munich, Germany) using the BlueBAG™ and BodyFIX^®^ immobilization system (Elekta, Stockholm, Sweden), hereafter referred to as the vac bag. The CT image dataset was then sent to Pinnacle^3®^ 9.10 (Koninklijke Philips N.V., Amsterdam, the Netherlands) and a 4 × 4 cm^2^ 360° arc was planned and delivered to the phantom. A dose calculation grid that encompasses the whole patient was used with a resolution of 2.5 mm.^24^ 1 × 1 cm^2^ contours were added allowing for calculation of skin dose at points of interest. These contours were mirrored laterally, to correspond to five horizontal regions, *g*_*i*_, in the ant/post directions, as shown in Fig. 1, that broadly represents anterior, posterior, and lateral regions as well as regions in between with and without vac bag involvement.

Eight pieces of Garchromic™ EBT^3^ film (Ashland Specialty Ingredients, Bridgewater, NJ, USA) were used to measure the surface dose at the eight locations specified in the treatment plan when the immobilized CIRS phantom was irradiated using the 4 × 4 cm^2^ arc. Care was taken to minimize air gaps between the film and phantom surface, by cutting sufficiently small (1 × 1 cm^2^) pieces of film. After irradiation, the films were scanned on a Perfection^®^ V850 Pro scanner (Epson^®^, North Ryde, NSW, Australia) and analyzed in the red and green channels through ImageJ (NIH, USA) using a rational dose calibration curve according to the methods of Micke et al.^25^

Skin dose correction factors ($c_{g_{i}}$) were then defined for each of the horizontal regions *g*_*i*_ shown in Fig. 1, as the ratio of the mean surface dose measured using film, ${\bar{D}}_{\mathrm{film},g_{i}}$, to the mean surface dose calculated by the TPS of the film contour, ${\bar{D}}_{\mathrm{TPS},g_{i}}$

(1) $$c_{g_{i}}=\;\frac{{\bar{D}}_{\mathrm{film},g_{i}}}{{\bar{D}}_{\mathrm{TPS},g_{i}}}$$

The effective depth of measurement of EBT film is 0.2 mm (i.e., within the film/skin contour) and PDD correction factors can be applied to film readings for a particular depth of interest based on the buildup PDD.^7^ However, the magnitude of these corrections is largely diminished when surface film is also exposed to exit dose from another beam or opposite side of a VMAT arc.^7^ The corrections are also field size‐dependent^7^ and application is confounded by the apparent disagreement in the literature regarding the true value of surface dose.^1^, ^7^, ^26^ In addition, the experimental setup in this study also concerns skin dose enhancements effects, further complicating what, if any, depth corrections are to be applied to film. As such, the uncertainty in ${\bar{D}}_{\mathrm{TPS},g_{i}}\;$ accounts for the range of dose values in the buildup across its volume. The principle of using surface measurements for comparison to deeper skin dose follows that of other studies in the literature.^1^, ^12^, ^20^

### 2.B Correction of skin dose calculations

Clinically realistic VMAT treatments planned for delivery to a humanoid phantom were used to exemplify the use of skin dose correction factors to improve the accuracy of skin dose calculations under more complex treatment conditions.

**Figure 2:**
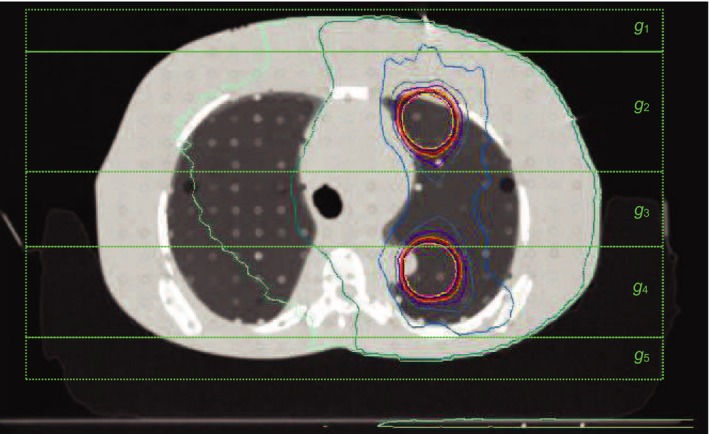
Vac bag immobilized ART phantom planned with SBRT delivery to anterior and posterior lung targets shown with composite dose from both plans. Calculation grid regions *g*_*i*_ are shown.

An Alderson Radiation Therapy (ART) phantom (Supertech^®^, Elkhart, IN, USA) was immobilized and CT scanned, using the same type of immobilization devices as were used for the simple CIRS phantom. The resulting image dataset was exported to the Pinnacle TPS and used to plan two test VMAT treatments (see Fig. 2). Since high‐grade skin toxicity has been observed for SBRT lung treatments, 3 cm targets were contoured in the anterior and posterior lung positions and given a hypofractionated SBRT prescription of 54 Gy in 3 fractions, according to recommendations of RTOG 0618.^27^ Organs at risk were also contoured in a realistic manner and given constraints according to RTOG 0618. Like the CIRS phantom reference measurements, the ART phantoms skin structure was created using the established method of contouring a 5‐mm thick ring of tissue beneath the external surface contour.^1^, ^20^

After the VMAT treatment plans had been created and the resulting doses calculated, the skin doses calculated by the TPS were modified according to the following procedure. The calculation grid used for the VMAT dose calculations was restricted to cover only one of the regions, *g*_1_ to *g*_5_ (as shown in Fig. 2), at a time. The number of monitor units (MU) used in the treatment were scaled with the correction factor identified using the film measurements on the CIRS phantom, for the region being calculated. The volumes of skin exposed, $v_{x,g_{i}}$, to doses of x Gy (in this case, 2.6, 6.5 and 13 Gy, corresponding to a Grade 1, 1–2, and 2–3 reactions, respectively, for 3 fraction SBRT^28^, ^29^) were recorded and summed over the calculation regions *g*_*i*_ to calculate $V_{x}$ according to eq. (2). For example, if the region *g*_*i*_ had a correction factor of $c_{g_{i}}$ and the treatment had a prescription of y MU then for calculation of grid *g*_*i*_, the number of monitor units was changed to $c_{g_{i}}\times \;y$ and $v_{x,g_{i}}$ was extracted accordingly.

(2) $$V_{x}=\;\sum_{i=1}^{n}v_{x,g_{i}}$$

### 2.C Verification of corrected skin dose calculations

To determine whether the skin dose correction factors improved TPS skin dose estimates of VMAT treatments delivered to the humanoid ART phantom, additional film measurements were used to evaluate the surface doses delivered to the ART phantom by the planned VMAT treatments. A CIRS phantom was selected for the derivation of $c_{g_{i}}$ due to its simplicity and popularity and an ART phantom for verification of this study's method due to its accuracy as a human surrogate.

Film pieces were fixed to the phantom surface while treatment was delivered. The measured film doses were compared against appropriate dose contours calculated in the VMAT treatment plans, with and without the use of the skin dose correction employed in this study. The method employed in this study was also compared to the results from the method shown by Wang et al., whereby the external patient contour is expanded by 2 cm and extended to include the immobilization device^1^ and the method demonstrated by Lee et al. where the vac bag is contoured in the TPS.^20^

In addition to comparisons of film contour dose, values of $V_{x}$ calculated according to eq. (2) were also compared to the uncorrected TPS dose volume values as well values based on the methods of Wang et al.^1^ and Lee et al.^20^ These were in turn compared to dose volume values derived from the in vivo film measurements of the SBRT lung treatments on the ART phantom, in order to surmise which method was the most accurate.

## 3 RESULTS

Values of $c_{g_{i}}$ calculated from the example 4 × 4 cm^2^ reference arc are listed in Table 1. Surface dose measurements for the reference arc were in agreement with anterior and lateral TPS calculations within calculated uncertainties. However, at posterior points, measured surface doses were as much as double those calculated by the TPS.

**Table 1:** Calculation of grid region correction factors, $c_{g_{i}}$, from reference arc exposure on the CIRS phantom (1 SD)

*g*_*i*_	${\bar{D}}_{\mathrm{TPS},g_{i}}$	${\bar{D}}_{\mathrm{film},g_{i}}$	$c_{g_{i}}$
*g*_1_	3.67 ± 1.05	3.88 ± 0.03	1.00
*g*_2_	3.26 ± 0.89	3.35 ± 0.04	1.00
*g*_3_	2.95 ± 0.78	3.63 ± 0.04	1.00
*g*_4_	2.90 ± 0.84	5.42 ± 0.07	1.87 ± 0.56
*g*_5_	2.96 ± 1.34	6.55 ± 0.09	2.21 ± 1.03

Mean measured film dose from SBRT exposure on the ART phantom for each region *g_i_* was compared to TPS predictions as well as corrected predictions based on the method by Wang et al.^1^ and Lee et al.^20^ as well as the method employed in this study through the use of grid region correction factors $c_{g_{i}}$. Skin dose values are listed in Table 2 and show no significant difference between measured and calculated dose for *g*_1_, *g*_2_, and *g*_3_ but for *g*_4_ and *g*_5_, the dose calculated through the method employed in this study was much closer to the measured film values than the raw TPS predictions. The values calculated from the Wang et al. and Lee et al. methods were found to be equivalent to the uncorrected TPS values. For the anterior lung plan, the difference between measured and TPS values changed from −45.8% to 2.04% and from −53.5% to 2.08% for *g*_4_ and *g*_5_, respectively, by applying grid region correction factors. *g*_5_ in the posterior plan did not see any notable improvements but the difference for *g*_4_ changed from −47.8% to −2.35%.

**Table 2:** Measured skin doses for each region *g*_*i*_ on the ART phantom for the two SBRT lung plans compared to corrected and uncorrected TPS predictions (1 SD)

Plan	*g*_*i*_	Measured dose (Gy)	TPS dose (Gy)	Corrected TPS dose (Gy)
Anterior lung	*g*_1_	2.60 ± 0.03	3.39 ± 1.43	3.39 ± 1.43
	*g*_2_	3.21 ± 0.02	3.46 ± 1.63	3.46 ± 1.63
	*g*_3_	4.45 ± 0.02	3.41 ± 1.11	3.41 ± 1.11
	*g*_4_	3.84 ± 0.03	2.10 ± 1.12	3.93 ± 2.10
	*g*_5_	6.68 ± 0.05	3.11 ± 1.12	6.87 ± 2.48
Posterior lung	*g*_1_	2.28 ± 0.02	2.38 ± 1.19	2.38 ± 1.19
	*g*_2_	2.66 ± 0.02	3.29 ± 1.41	3.29 ± 1.41
	*g*_3_	3.94 ± 0.02	3.26 ± 0.82	3.26 ± 0.82
	*g*_4_	7.59 ± 0.05	3.97 ± 1.67	7.42 ± 3.12
	*g*_5_	6.18 ± 0.09	3.89 ± 1.44	8.60 ± 3.18

Skin values of *V*_2.6 Gy_, *V*_6.5 Gy_ and *V*_13 Gy_ for the two SBRT lung plans delivered on the ART phantom are shown in Table 3. The “measured” values were calculated based on film sampling taken during treatment delivery and are compared against the four methods of skin dose determination: the raw TPS predictions, external patient contour expansion shown by Wang et al.,^1^ tissue‐equivalent contouring of vac bag shown by Lee et al.^20^ and the method in this study utilizing $c_{g_{i}}$. As expected from the results in Table 2, the methods demonstrated by Wang et al. and Lee et al. had little to no effect in the Pinnacle TPS, while the methodology employed in this study made substantial differences to calculations of skin dose volumes.

**Table 3:** Measured skin dose volumes compared to different methods of calculation/correction from verification of SBRT lung treatments on an ART phantom

Plan	Method	*V*_2.6 Gy_ (cc)	% diff	*V*_6.5 Gy_ (cc)	% diff	*V*_13 Gy_ (cc)	% diff
Anterior lung	Measured	87.22		32.69		0.26	
	TPS	61.64	−29.3%	12.94	−60.4%	0.04	−84.6%
	Wang et al.	61.64	−29.3%	12.94	−60.4%	0.04	−84.6%
	Lee et al.	62.11	−28.8%	13.62	−58.3%	0.04	−84.6%
	This study	89.18	2.2%	33.72	3.2%	0.35	30.8%
Posterior lung	Measured	88.77		37.63		12.13	
	TPS	65.19	−26.6%	15.51	−58.8%	0.14	−98.8%
	Wang et al.	65.19	−26.6%	15.51	−58.8%	0.14	−98.8%
	Lee et al.	65.21	−26.5%	15.31	−59.3%	0.13	−98.9%
	This study	90.38	1.8%	38.85	3.2%	14.86	22.5%

## 4 DISCUSSION

Using the CIRS phantom, values of $c_{g_{i}}$ for five dose calculation regions were calculated from a reference arc exposure. Disagreement between skin dose calculations and measurements (i.e., $c_{g_{i}}\neq \;1.0$) in the absence of adjacent immobilization reported elsewhere^30^ was not observed in this study. All anterior and lateral film measurements were within the uncertainties of TPS predictions. Posteriorly, the TPS was not able to accurately consider the vac bag and any buildup effect possibly produced by the couch top was diminished by the “air gap” between the couch and the phantom. The posterior skin dose discrepancy and subsequent values of $c_{g_{i}}$ were in agreement with similar measurements seen in the literature.^9^, ^10^, ^11^ Uncertainties were large due to the dose gradients at the skin surface.

For the SBRT deliveries on the ART phantom, substantial improvement was observed for the simple comparison of film dose values where the correction was applied through the use of $c_{g_{i}}$ factors. While the uncorrected TPS dose values (and subsequent values calculated from the methods of Wang et al. and Lee et al.) were almost half of that measured, grid region correction was able to bring the TPS values to within 3% of the measured values, demonstrating and verifying the accuracy in using reference values for subsequent treatment plans on different patients where similar immobilization is utilized. In this case, the vac bag shapes were different due to the different phantoms and agreement was still exhibited for 9 out of 10 grid regions considered.

This agreement between the corrected TPS and measured values was further demonstrated when skin dose volumes were calculated. For skin receiving at least a Grade 1 reaction, the TPS underestimated for both anterior and posterior SBRT plans by up to 29.3%, while the method in this study improved this calculation by as much as 27% to be within 2% of the measured value. An even greater improvement in accuracy of between 55% and 76% was seen for the volume of skin receiving Grade 1–2 and 2–3 reactions (a greater difference and variability was observed for the Grade 2–3 values due to smaller volumes). This demonstrates that the gross underestimations of skin dose can be easily improved through the utilization of the correction methods shown in this study. This will greatly assist clinicians in accurately predicting medium and high‐grade skin reactions in patients and thus avoid, where possible, treatment planning practices that could cause severe skin toxicity. If the method is applied to a particular plan and it indicates undesirable skin toxicity, a tighter dose constraint to skin can be applied without fear of adverse effect to plant conformity.^20^

The method demonstrated by Wang et al. to improve skin dose calculation in the presence of immobilization devices appears to be specific to the Eclipse TPS^1^ due to the way Eclipse uses the external patient contour to define the extent of the calculation volume. For the SBRT plans in this study, the external patient contour was expanded and the dose recalculated in accordance with their method without any effect to the dose distribution in Pinnacle. In some cases, depending on the quality of the model, external contour expansion may not make the necessary accuracy improvements to skin dose volume calculations in Eclipse, in which case, the methods demonstrated in this study could be applied to improve skin dose accuracy.

The method shown by Lee et al. also seems to be specific to their unnamed pencil beam TPS, and perhaps more so to IMRT as their study only considered the case of a beam passing through both vac bag and couch.^20^ Similarly for Eclipse in the Wang et al. study, the TPS used by Lee et al. may not routinely consider volumes outside of the patient contour, thus it was vital for them to include a contour, so any skin enhancement was accounted for. Pinnacle sidesteps this issue by utilizing all CT dataset information in beam transport calculations unless overridden, hence no difference was seen when testing Lee et al.'s method in this study. The correction method demonstrated in this study is applicable for VMAT and instead of relying on the TPS surface dose calculation applied differently,^1^, ^20^ corrects it directly based on physical measurement.

The demonstrated method for more accurately estimating skin dose in the presence of immobilization is flexible and can be made more or less precise through choice of grid regions, *g*_*i*_. The immobilization scenario in this study utilized five grid regions corresponding to five scenarios of immobilization/couch involvement, but a different immobilization scenario is expected to require a different number of grid regions. For example, the user may choose, instead of dose grid regions in rows, to add many small square grids all around the patient contour, so that $c_{g_{i}}$ are asymmetrical. Alternatively, the method can be used in the same way but for a thermoplastic mask/sheet or any other immobilization device where a skin dose enhancement is expected. Verification of the use of $c_{g_{i}}$ derived using the common CIRS phantom also suggests other departments can use their CIRS phantom (or equivalent) to derive correction factors for their own immobilization systems/beam qualities/planning systems. The method can be applied through the following simple steps:

1. Immobilize and CT scan a simple anthropomorphic phantom with the immobilization equipment of interest
2. Create a “reference” open field arc on the phantom CT dataset in the TPS
3. Affix film pieces to the phantom surface corresponding to the number and location of grid regions desired (see Fig. 1 as an example)
4. Deliver the reference field to the immobilized phantom
5. Use eq. (1) to calculate grid region correction factors
6. On subsequent patients with the same immobilization, define appropriate *g*_*i*_ in the TPS and use the reference values of $c_{g_{i}}$ through eq. (2) to more accurately calculate skin dose volumes

## 5 CONCLUSION

Measurements with film demonstrated that, if uncorrected, surface dose levels can be as much as double the Pinnacle TPS calculation where the impact of vac bag and couch is the greatest. This has a great effect in predicting the volume of skin that will present with a severe reaction. A simple method, whereby the TPS calculation of skin dose is corrected by scaling dose in calculation grid regions based on reference measurements, was verified through application on SBRT lung plans in an anthropomorphic phantom. The method employed was able to improve the skin dose volume predictions by up to 76% and, for NCI Grade 1–2 reactions, correct the TPS values to within 3% of the measured values. This method is straightforward, can be applied to most immobilization systems and has the potential to assist in predicting and avoiding skin reactions, especially from hypofractionated VMAT treatments.

## CONFLICTS OF INTEREST

The authors have no other relevant conflicts of interest to disclose.
